# Effective Antiviral Therapy Improves Immunosuppressive Activities in the Immune Microenvironment of Hepatocellular Carcinoma by Alleviating Inflammation and Fibrosis

**DOI:** 10.1002/cam4.70459

**Published:** 2024-12-10

**Authors:** Zhu‐tao Wang, Ruo‐yu Guan, Wei Gan, Zhang‐fu Yang, Bao‐ye Sun, Jing‐fang Wu, Dai Zhang, Guo‐qiang Sun, Xu‐kang Gao, Jin‐long Huang, Gao Liu, Cheng Zhou, Jian Zhou, Jia Fan, Yong Yi, Bo Hu, Shuang‐Jian Qiu

**Affiliations:** ^1^ Department of Liver Surgery and Transplantation, Zhongshan Hospital Fudan University Shanghai China; ^2^ Liver Cancer Institute and Biomedical Research Center, Zhongshan Hospital Fudan University Shanghai China; ^3^ Department of General Surgery, Zhongshan Hospital Fudan University Shanghai China

**Keywords:** antiviral therapy, hepatocellular carcinoma, immune microenvironment, inflammation and fibrosis

## Abstract

**Background and Aims:**

The immune microenvironment (IME) plays a crucial role in the progression of hepatocellular carcinoma (HCC). In HCC, the IME is often compromised by hepatitis B virus (HBV) infection, chronic inflammation, and fibrosis. Both antiviral therapy (AVT) and the alleviation of inflammation and fibrosis (AIF) have been shown to improve prognosis. However, the relationship among the IME of HCC, AVT, and AIF remains unclear.

**Methods:**

A total of 140 and 110 primary HBV‐related HCC patients were enrolled as training and validation sets, respectively, to establish a HCC‐immune microenvironment score (H‐IME score). Immunohistochemistry was performed to assess the number of granzyme B+ (GrB+) and Foxp3+ cells, as well as the expression of CTLA‐4, PD‐1, LAG3, TIGIT, TIM3, and VISTA. Another cohort consisting of 114 recurrent HBV‐related HCC patients with paired primary and recurrent tissues was used to study the relationship among the IME of HCC, AVT, and AIF.

**Results:**

The H‐IME score, including GrB, Foxp3, CTLA‐4, PD‐1, LAG3, and TIGIT, was established to evaluate the IME. A higher H‐IME score indicates stronger immunosuppressive activities. Both AVT and AIF were found to inhibit immunosuppressive activities in the IME. Compared to primary tumors, the H‐IME scores of recurrent tumors in the effective AVT group (e‐AVT, classified by HBV DNA) with AIF decreased, while the scores increased in the non‐AVT group without AIF.

**Conclusions:**

The IME of HCC is closely related to AVT and AIF. e‐AVT can enhance anti‐tumor activities in the IME by alleviating inflammation and fibrosis.

## Graphical abstract

In this study, we utilized training and validation cohort to establish a scoring system (hepatocellular carcinoma (HCC) immune microenvironment (H‐IME) score) for IME in HCC, taking number and functional status of infiltrating activated effector cells and inhibitory cells into account, firstly. H‐IME score was then used in study the relationship among antiviral therapy (AVT), alleviation of inflammation and fibrosis (AIF) and IME of HCC in patient's paired primary and recurrent HCC specimens. We propose a new trilogy of ‘effective‐AVT‐AIF‐ameliorative IME’, suggesting early initiation of AVT should be considered for HBV‐HCC patients.

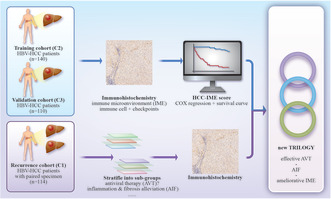

## 1 Introduction

The immune microenvironment (IME) refers to the microenvironment formed by immune cells and their products, which plays a crucial role in cancer development [1]. Comparative research on the IME of recurrent tumors (RT) and primary tumors (PT) provides valuable insights into tumor recurrence mechanisms, the impact of the host organmicroenvironment on tumors, and the effects of drug therapy after tumor resection. RNA sequencing found that compared to PT, more changes happen in infiltrating cells, but not tumor cells, indicating the importance of the microenvironment in tumor recurrence [2]. In general, the IME in RT exhibits stronger immunosuppressive activities than that in primary tumors. For example, recurrent glioblastoma, osteosarcoma, head and neck squamous cell carcinoma, ovarian cancer, and nasopharyngeal carcinomas have been reported to have a more immunosuppressive IME, characterized by reduced effector cells infiltration and upregulated expression of immune checkpoints [3, 4, 5, 6, 7, 8]. Liver metastasis shows increased infiltration of inhibitory immune cells such as MRC1 + CCL18+ macrophages, SPP1+ macrophages, and regulatory T cells (Tregs) compared to primary colorectal tumors [9]. Similarly, there are more inhibitory cells (Tregs and M2 macrophages) in malignant pleural effusion than PT [10].

Hepatocellular carcinoma (HCC) is a leading cause of cancer‐related mortality worldwide and has a high recurrence rate, with approximately 80% of cases experiencing postoperative recurrence [11]. Comparative studies between HCC RT and PT have shown that the gene expression signature of primary HCC is similar to that of corresponding metastasis [12]. Furthermore, key genes involved in the process of recurrence and metastasis, such as ARID1A, VCAM1, and CDK14 mutations, have been identified [13]. Significant imbalances in the inflammatory IME in para‐carcinoma tissue have been shown to promote the metastasis and recurrence of liver cancer [14]. Immune cells in early recurrent HCC exhibit a more naïve, hypofunctional phenotype, while tumor cells demonstrate a stronger immune escape ability, indicating distinct characteristics of the IME [15, 16].

HCC is a typical inflammation‐related tumor, with hepatitis B virus (HBV) infection being the primary cause of HCC in Asia [11]. HBV directly affects the immune system's function by creating a chronic inflammatory microenvironment and disrupting the stability of the host genome through various mechanisms [17]. Carcinoma tissues in HBV‐related HCC (HBV‐HCC) are characterized by an abundance of PD‐1 or CTLA‐4 expressing Tregs compared to HCC without HBV infection [18, 19]. Mice with high HBV loads exhibit a significant increase in PD‐1+ cells [20]. Antiviral therapy (AVT) has been shown to effectively prevent the occurrence of HCC, reduce the recurrence rate, and improve overall survival (OS) [21, 22], while non‐AVT was independent risk factor for HBV‐HCC [23]. Our previous research has demonstrated that AVT can alleviate inflammation and fibrosis in HBV‐HCC patients, leading to improved prognosis [24]. Sequencing data also found that AVT attenuates percentage of hyperactivated effector immune cells [25]. However, the relationship among the IME in HCC, AVT, and the alleviation of inflammation and fibrosis (AIF) remains unclear.

In this study, a scoring system called the HCC‐IME (H‐IME score) for evaluating the IME was established based on two cohorts, with 140 and 110 primary HBV‐HCC patients, respectively. Immunohistochemistry was performed to assess the number of granzyme B+ (GrB+) and Foxp3+ cells, as well as the expression of CTLA‐4, PD‐1, LAG3, TIGIT, TIM3, and VISTA. Additionally, we collected a cohort of 114 recurrent HBV‐HCC patients with preserved samples of paired primary and recurrent carcinoma and para‐carcinoma tissues to investigate the relationship among the IME of HCC, AVT, and AIF. Our findings demonstrate that the IME of HCC is closely associated with AVT and AIF. Effective AVT can inhibit immunosuppressive activities in the IME by alleviating inflammation and fibrosis. A new trilogy of “e‐AVT‐AIF‐ameliorative IME” was proposed.

## 2 Material and Methods

### 2.1 Patients and Cohorts

A total of 498 patients were selected from 2243 consecutive patients who underwent re‐hepatectomy for recurrent HCC between January 2010 and December 2019 at the Liver Cancer Institute, Zhongshan Hospital, Fudan University, according to the inclusion and exclusion criteria in our previous research [24]. There are 114 patients whose paired recurrent and primary carcinoma and para‐carcinoma tissues were well preserved in the cohort, namely cohort C1.

Two additional cohorts, named C2 and C3, were established for this study. These cohorts included a total of 140 and 110 patients, respectively, who underwent radical resection for primary HCC at the Department of Liver Surgery and Transplantation, Zhongshan Hospital (Shanghai, China) in 2012 and 2008. All patients had tested positive for hepatitis B surface antigen (HBsAg) for more than 3 months.

The study was approved by the Clinical Research Ethics Committee of Zhongshan Hospital, and written informed consent was obtained from all participants.

### 2.2 Immunohistochemistry and Quantitation

Immunohistochemistry and quantitation were performed following standard protocols described in our previous publication [26]. After baking at 60° centigrade for 1 h, tissue microarray (TMA) sections were de‐paraffinized and rehydrated through xylene and graded ethanol, respectively. Sections were heated in Tris‐EDTA solution for antigen retrieval for 5 min. TMA were incubated with antibodies at 4° centigrade overnight and secondary antibodies at room temperature for 30 min. and then detected by treatment with diaminobenzidine chromogen for 3 min. Immune cells analyzed included Tregs (Foxp3+, CST, 98377s) and GrB+ (CST, 46890s) cells. The expression of checkpoints, namely CTLA‐4 (abcam, ab237712), PD‐1 (abcam, ab52587), LAG3 (abcam, ab209236), TIGIT (abcam, ab243903), TIM3 (abcam, ab241332), and VISTA (CST, 54979s), was also assessed.

For Foxp3 and GrB measurements, the average number of positive cells in five random images from each immunostained HCC section was recorded using a microscope (magnification, ×400). The number of Tregs and GrB+ cells was classified as high (1 point) if the number of positive cells was equal to or higher than the median of the series, and low (0 point) if it was below the median.

The expression levels of the six checkpoints were recorded as the ratio of positive cells to all immune cells in the field (magnification, ×400). The scoring of checkpoint expression was defined as positive (1 point) if the ratio was equal to or higher than 5% for CTLA‐4 and PD‐1, 1% for LAG3, TIGIT, TIM3, and VISTA, and negative (0 point) if the ratio was below the corresponding cutoff value.

### 2.3 Definition of Antiviral Therapy and Its Therapeutic Effect

AVT was defined as the consecutive and regular use of at least one kind of nucleos(t)ide analogues for more than 3 months, as previously described [24]. In primary and secondary surgery, the serum HBsAg level was measured within a week before the hepatectomy. The effective AVT (e‐AVT) group was defined as patients achieving HBV DNA suppression (HBV DNA < 2000–4000 IU/mL (HBeAg‐positive) or 2000 IU/mL (HBeAg‐negative) for Peg‐IFN, or < 60 IU/mL for entecavir and tenofovir) at recurrent hepatectomy, while non‐effective AVT (ne‐AVT) group referred to patients who failing to reach HBV DNA suppression [27].

### 2.4 Evaluation of Inflammation Level and Fibrosis Degree

Surgical specimens from hepatectomy were fixed, stained with hematoxylin and eosin, and assessed by two pathologists blind to clinical data independently. The Batts–Ludwig scoring system (GS score, G for inflammation and S for fibrosis) was used to evaluate the level of inflammation and fibrosis based on histopathology in non‐cancerous liver tissues [28], as described in a previous study [24]. Patients in the AIF group were defined as having a lower G or S score in the recurrent surgery compared to the primary surgery. Patients in the non‐AIF group had a stable or higher G or S score compared to the primary surgery. In the C1 cohort, there were no cases where inflammation improved and fibrosis progressed or inflammation progressed and fibrosis improved.

### 2.5 Follow‐Up Protocol

All patients were followed up every 3 months in the first 2 years and every 6 months until the end point (December 2020) or loss of follow‐up, after liver resection. Details were described in the aforementioned paper [24]. Recurrence within 2 years after primary hepatectomy was defined as early recurrent tumor (e‐RT), while recurrence after 2 years was defined as late recurrent tumor (l‐RT).

### 2.6 Statistical Analysis

Continuous variables were presented as means ± standard deviations, while categorical variables were presented as numbers. All statistical tests were two‐sided, and a *p*‐value less than or equal to 0.05 was considered statistically significant.

The Cox proportional hazard model was applied to identify significant factors related to patient prognosis. Only variables that were significant in the univariate Cox model were included in the multivariate Cox model. The cumulative OS and recurrence‐free survival (RFS) rates were analyzed using the Kaplan–Meier method and assessed with the log‐rank test. Normality test was performed by Kolmogorov–Smirnov test. Paired *t*‐tests were used for quantitative values conforming to normal distribution, while Wilcoxon paired tests for other quantitative values. Chi‐square test was employed for categorical variables. Statistical analysis was conducted using RStudio 1.2.5033 (R 4.2.1) and GraphPad Prism 8.3.0.

## 3 Results

### 3.1 Clinicopathological Characteristics of the Patients

The study comprised three cohorts: C2 cohort and C3 cohort served as the training and validation cohorts, respectively, for exploring the IME scoring system‐H‐IME score. C2 cohort included a total of 140 participants (115 males and 25 females), while C3 cohort included 110 participants (99 males and 11 females). The C1 cohort comprised 114 patients (98 males and 16 females), with an average age of 53.3 at the time of the initial surgery. C1 cohort was designed to study the IME of carcinoma and para‐carcinoma tissue in HBV‐HCC and its changes from PT to RT, using H‐IME scoring system. In the C1 cohort, 85 (74.6%) patients received AVT, while the remaining 29 (25.4%) participants were categorized into the non‐AVT group. Among the patients in the C1 cohort, 61 (53.5%) cases experienced an AIF, while 53 (46.5%) cases were classified into the non‐AIF group.

**FIGURE 1:**
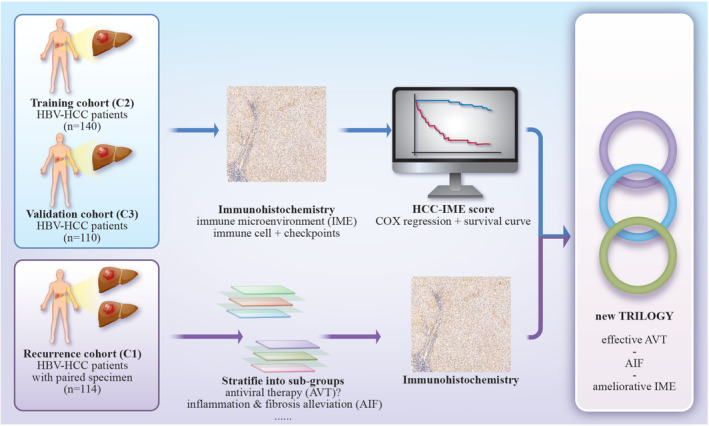
Graphical table of contents. In this study, we utilized training and validation cohort to establish tumor immune microenvironment (H‐IME) score for IME in HCC, taking number and functional status of infiltrating activated effector cells and inhibitory cells into account, firstly. H‐IME score was then used in study the relationship among AVT, AIF and IME of HCC in patient's paired primary and recurrent HCC specimens. Effective AVT (based on changes in HBV DNA load) can stabilize or even reverse level of inflammation and fibrosis in HCC, leading to enhanced anti‐tumor activities in the IME. We propose a new trilogy of ‘e‐AVT‐AIF‐ameliorative IME’, suggesting early initiation of AVT should be considered for HBV‐HCC patients.

**FIGURE 2:**
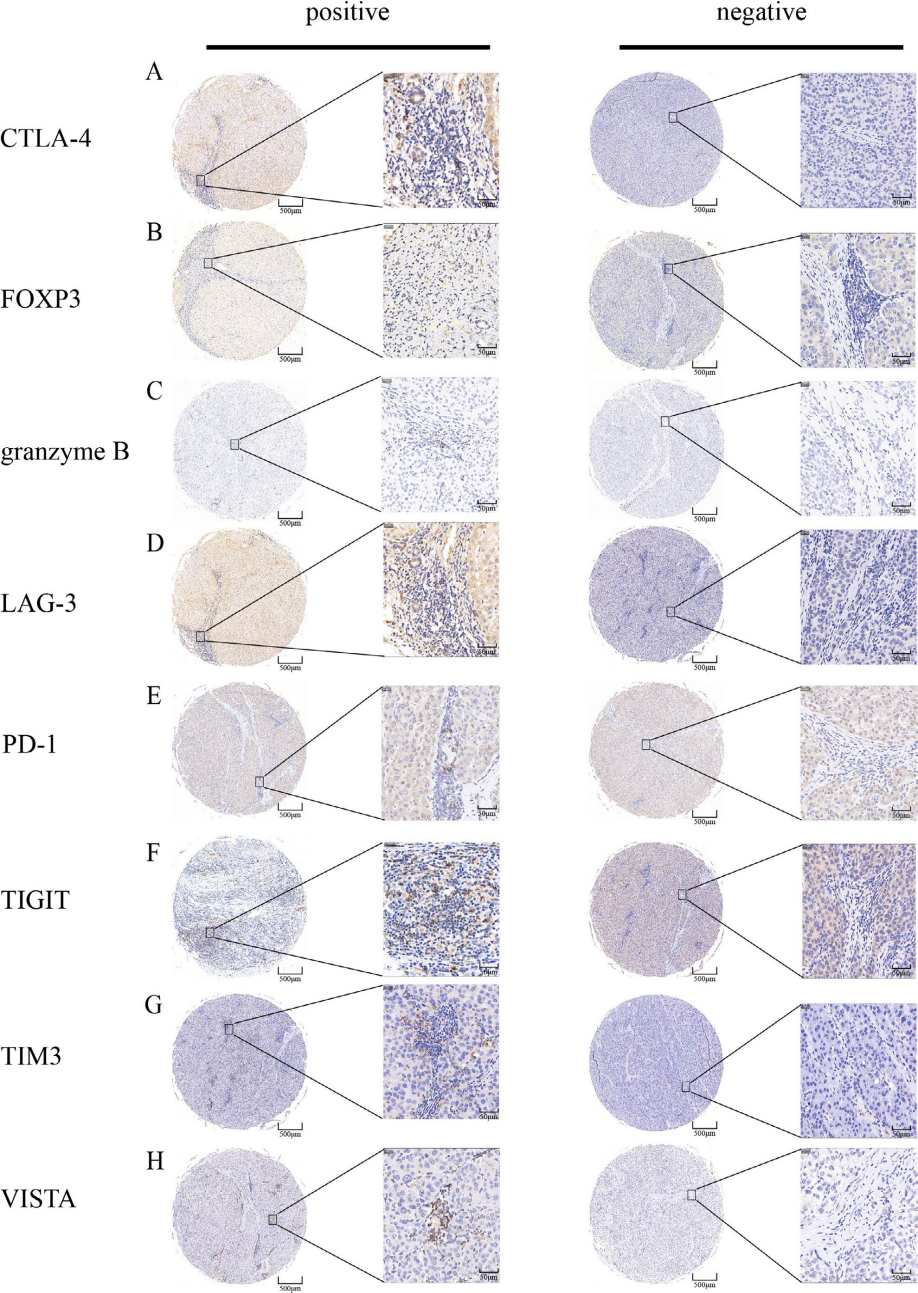
Representative immunohistochemical staining of CTLA‐4 (A), FOXP3 (B), granzyme B (C), LAG‐3 (D), PD‐1 (E), TIGIT (F), TIM3 (G), VISTA (H).

Table 1 presents the clinicopathological data of the participants in the C2, C3, and C1 cohorts at the initial hepatectomy. Supplementary demographic information of C1 cohort is listed in Table S1. The difference in microvascular invasion (MVI) among the three cohorts could be attributed to the fact that recurrence occurred in all patients in the C1 cohort, but not in all patients in the C2 or C3 cohorts. MVI has been reported as a major risk factor for recurrence [29]. The difference in MVI between the C2 and C3 cohorts was not statistically significant, with a *p*‐value of 0.1279. The most common side‐effect of AVT is liver and renal injury [30], while there are no significantly differences observed between AVT and non‐AVT group (Table S2). Figure 1 demonstrates the graphical Table of contents. Figure 2 shows typical photos of immunohistochemistry in carcinoma tissue.

**TABLE 1:** Comparison about the clinical and pathological characteristics at initial stage in three cohorts.

	C1 cohort (*n* = 114)	C2 cohort (*n* = 140)	C3 cohort (*n* = 110)	*p*
Gender (male/female)	98/16	115/25	99/11	0.2107
Age (years)	53.3 ± 8.9	52.7 ± 9.7	50.5 ± 10.3	0.0708
TBIL (μmol/L ± SD)	13.35 ± 17.6	12.0 ± 4.6	13.4 ± 5.9	0.5136
ALB (g/L ± SD)	40.79 ± 3.31	40.3 ± 2.9	40 ± 4	0.3395
ALT (U/L ± SD)	76.61 ± 291.50	39.1 ± 27.5	42.1 ± 20.8	0.1516
PT (S ± SD)	12.30 ± 0.79	12.5 ± 0.8	12.4 ± 1.1	0.3205
AFP (< 20 ng/mL/≥ 20 ng/mL)	55/59	44/66	53/87	0.2244
Tumor number (single/multiple)	98/16	108/32	84/26	0.1298
Tumor size (< 3 cm/≥ 3 cm)	35/79	34/106	20/90	0.0928
Tumor differentiation (1,2/3,4)	78/36	83/57	79/31	0.0923
MVI (no/yes)	33/81	81/59	74/36	< 0.0001****
AVT (no/yes)	29/85	——	——	——
e‐AVT (no/yes)	8/77	——	——	——
AIF (no/yes)	53/61	——	——	——

Abbreviations: AIF, alleviated inflammation and fibrosis; ALB, albumin; ALT, alanine; AVT, anti‐viral therapy; e‐AVT, effective AVT; MVI, microvascular invasion; PT, prothrombin time; TBIL, total bilirubin.

****p value < 0.0001.

### 3.2 Establishment and Validation of the H‐IME Score

**FIGURE 3:**
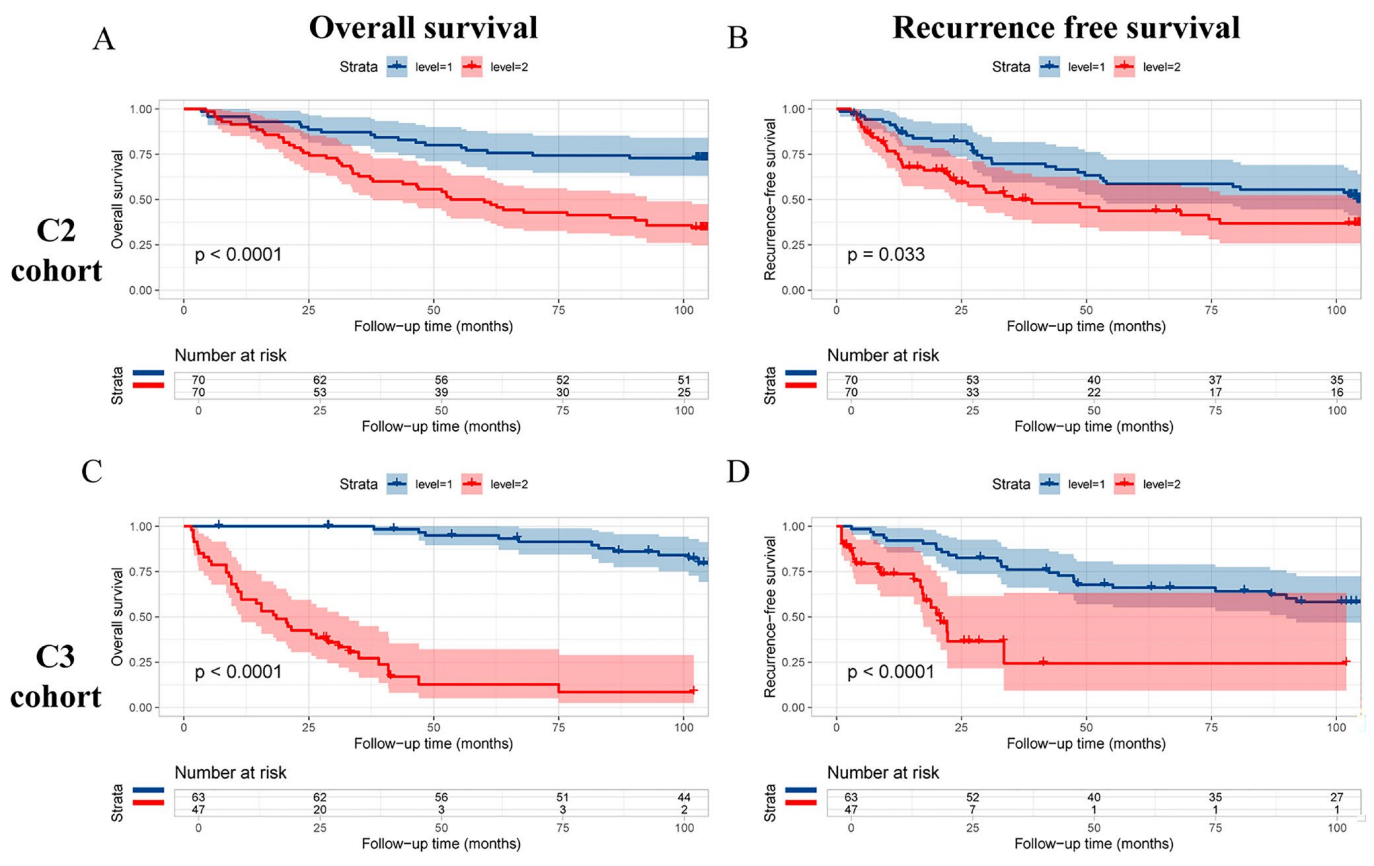
Comparison of the prognostic outcomes between high and low level of H‐IME score. (A) OS in C2 cohort; (B) RFS in C2 cohort; (C) OS in C3 cohort; (D) RFS in C3 cohort.

In the univariate Cox analysis of the C2 cohort (training set), GrB (0.21–0.69, *p* = 0.0016), Foxp3 (1.33–4.21, *p* = 0.0034), CTLA‐4 (3.10–11.73, *p* < 0.0001), PD‐1 (1.90–6.38, *p* = 0.0001), LAG3 (2.23–7.22, *p* < 0.0001), TIGIT (2.60–8.56, *p* < 0.0001), TIM3 (2.73–11.75, *p* < 0.0001), and VISTA (1.52–6.48, *p* = 0.0021) were significantly correlated with OS. In the multivariate Cox analysis, GrB (0.26–0.92, *p* = 0.0272), Foxp3 (1.00–3.56, *p* = 0.0496), CTLA‐4 (1.45–6.69, *p* = 0.0037), PD‐1 (1.01–3.84, *p* = 0.0455), LAG3 (1.12–4.00, *p* = 0.0215), and TIGIT (1.17–4.44, *p* = 0.0150) remained significant predictors of OS, with regression coefficients of −0.7, 0.6, 1.1, 0.7, 0.7, and 0.8, respectively (Table 2). Based on the Cox analysis, the H‐IME score can be summarized as follows: “H‐IME score = ‐0.7GrB + 0.6Foxp3 + 1.1CTLA‐4 + 0.7PD‐1 + 0.7LAG3 + 0.8TIGIT.” In the C2 cohort, the H‐IME score was proven to be effective in predicting prognosis. The 1‐, 3‐, and 5‐year OS rates were 91.4%, 62.9%, and 50% in the high H‐IME score group, and 95.7%, 87.1%, and 77.1% in the low H‐IME score group, respectively, with a *p*‐value less than 0.0001 (Figure 3). In the C3 cohort (validation set), the H‐IME score was also significantly correlated with both OS and RFS, with *p*‐values less than 0.0001 (Figure 3). Compared to tumor size and AFP, two traditional prognostic markers in HCC, H‐IME score showed larger area under curve (0.8079 in cohort C2, 0.8540 in cohort C3, Figure S1), indicating a better predictive ability.

**TABLE 2:** Univariate and multivariate Cox regression analysis for OS in T16.

	Uni‐COX		Mul‐COX	
	Exp.*β*	*p*	Exp.β	*p*
GrB	0.38 (0.21–0.69)	0.0016**	0.48 (0.26–0.92)	0.0272**
FOXP3	2.37 (1.33–4.21)	0.0034**	1.89 (1.00–3.56)	0.0496*
CTLA4	6.03 (3.10–11.73)	< 0.0001****	3.11 (1.45–6.69)	0.0037**
PD1	3.49 (1.90–6.38)	**0.0001**	1.97 (1.01–3.84)	0.0455*
LAG3	4.02 (2.23–7.22)	< 0.0001****	2.11 (1.12–4.00)	0.0215*
TIGIT	4.71 (2.60–8.56)	< 0.0001****	2.28 (1.17–4.44)	0.0150*
TIM3	5.67 (2.73–11.75)	< 0.0001****	1.89 (0.82–4.34)	0.1341
VISTA	3.13 (1.52–6.48)	0.0021**	1.99 (0.92–4.28)	0.0797

*p ≤ 0.05; **p < 0.01; ***p < 0.001; ****p < 0.0001.

### 3.3 H‐IME Score Is Higher in l‐RT Than in PT Without Intervention

Considering the significant role of HBV in the progression of HCC and the effect of AVT on IME, the non‐AVT group was selected to study the characteristics of IME in RT. In general, the IME of carcinoma tissue in RT was found to be more immunosuppressive than that in PT (H‐IME score: 2.33 vs. 1.57, *p* = 0.0037, Table 3). Based on the duration between PT and RT where cutoff value was 2 years, subjects were classified into e‐RT and l‐RT groups. No significant changes were observed in IME in e‐RT, while l‐RT showed a higher H‐IME score (2.44 vs. 1.16, *p* < 0.0001, Table 3), indicating a more repressive state. However, there were no notable differences in IME of para‐carcinoma tissue between RT and PT, regardless of the e‐RT or l‐RT group.

**TABLE 3:** Comparison between IME in RT and PT, without intervention.

		Carcinoma tissue		Para‐carcinoma tissue	
	Number	H‐IME score	*p*	H‐IME score	*p*
PT versus RT	114	2.33 ± 0.98 versus 1.57 ± 0.99	0.0037**	1.30 ± 1.25 versus 1.24 ± 1.21	0.8517
e‐RT versus PT	51	2.15 ± 1.00 versus 2.24 ± 0.95	0.8434	1.20 ± 1.18 versus 1.52 ± 1.24	0.5719
l‐RT versus PT	63	2.44 ± 0.99 versus 1.16 ± 0.79	< 0.0001****	1.37 ± 1.32 versus 1.07 ± 1.20	0.4877

**p < 0.01; ****p < 0.0001.

### 3.4 Both e‐AVT and AIF Can Mitigate the Immunosuppressive State in IME

The immunosuppressive state of IME in RT was less pronounced in the AVT group (H‐IME score: 1.22 vs. 1.77, *p* = 0.0009, Table 4) and more pronounced in the non‐AVT group (H‐IME score: 2.33 vs. 1.57, *p* = 0.0037, Table 4) compared to PT. Due to the presence of drug resistance in HBV AVT, the AVT group was further subdivided into the e‐AVT and ne‐AVT groups based on the level of HBV DNA load in peripheral blood. In the e‐AVT group, the IME in RT remained less immunosuppressive than that in PT (H‐IME score: 1.08 vs. 1.82, *p* < 0.0001, Table 4). Additionally, the IME of carcinoma tissue in the AIF group in RT was less repressive than in PT (H‐IME score: 1.16 vs. 1.94, *p* = 0.0001, Table 4), while the IME of RT in the non‐AIF group was more repressive than in PT (H‐IME score: 1.90 vs. 1.46, *p* = 0.0186, Table 4). In para‐carcinoma tissue, the IME of RT in the e‐AVT group or AIF group was less immunosuppressive than in PT.

**TABLE 4:** Effect of AVT and AIF on IME of recurrent HCC.

			Carcinoma tissue		Para‐carcinoma tissue	
	Number		H‐IME score	*p*	H‐IME score	*p*
Anti‐viral	85	AVT RT versus PT	1.22 ± 1.18 versus 1.77 ± 0.96	0.0009***	0.64 ± 1.10 versus 1.26 ± 1.18	0.0002***
	29	Non‐AVT RT versus PT	2.33 ± 0.98 versus 1.57 ± 0.99	0.0037**	1.30 ± 1.25 versus 1.24 ± 1.21	0.8517
	77	e‐AVT RT versus PT	1.08 ± 1.11 versus 1.82 ± 0.92	< 0.0001****	0.57 ± 1.07 versus 1.25 ± 1.19	0.0001***
	8	ne‐AVT RT versus PT	2.53 ± 1.08 versus 1.28 ± 1.28	0.1484	1.30 ± 1.27 versus 1.30 ± 1.16	0.9375
Inflammation and fibrosis	61	AIF RT versus PT	1.16 ± 1.22 versus 1.94 ± 0.91	0.0001***	0.59 ± 1.17 versus 1.37 ± 1.24	0.0003***
	53	Non‐AIF RT versus PT	1.90 ± 1.13 versus 1.46 ± 0.98	0.0186*	1.05 ± 1.14 versus 1.13 ± 1.12	0.7372

*p ≤ 0.05; **p < 0.01; ***p < 0.001; ****p < 0.0001.

### 3.5 E‐AVT Can Alleviate the Immunosuppressive State in IME by Reducing Inflammation and/or Fibrosis

Although e‐AVT did not shorten the duration between PT and RT, it was found to alleviate inflammation and fibrosis in RT. In both the e‐RT (*p* = 0.0012, Table 5) and l‐RT (*p* = 0.0019, Table 5) groups, a higher proportion of participants in the e‐AVT group showed a decrease in the GS score compared to the non‐AVT group.

**TABLE 5:** Correlation between AVT and AIF.

		e‐AVT	Non‐AVT	*p*
	Recurrence time	37/40	11/18	0.3882
	Early/late			
	Inflammation and fibrosis	52/25	5/24	< 0.0001****
	AIF/non‐AIF			
e‐RT	Inflammation and fibrosis	28/9	2/9	0.0005***
	AIF/non‐AIF			
l‐RT	Inflammation and fibrosis	24/16	3/15	0.0022**
	AIF/non‐AIF			

**p < 0.01; ***p < 0.001; ****p < 0.0001.

Among participants in the AIF group who received e‐AVT, the H‐IME score in both carcinoma tissue (H‐IME score: 1.01 vs. 2.01, *p* < 0.0001, Table 6) and para‐carcinoma tissue (H‐IME score: 0.45 vs. 1.35, *p* = 0.0001, Table 6) in RT showed a significant decrease compared to PT. In contrast, in the non‐AIF group who did not receive AVT, the immunosuppressive activity in IME increased in carcinoma tissue in RT (H‐IME score: 2.47 vs. 1.59, *p* = 0.0048, Table 6), while no significant difference was observed in para‐carcinoma tissue. Furthermore, the H‐IME score in RT was similar to that in PT in patients in AIF group who did not receive AVT (H‐IME score: 1.66 vs. 1.48, *p* = 0.4047. Table 6). Such phenomenon was observed in patients in non‐AIF who achieved e‐AVT (H‐IME score: 1.24 vs. 1.43, *p* = 0.3515).

**TABLE 6:** Effect of combination of AVT and AIF on IME of recurrent HCC.

			Carcinoma tissue		Para‐carcinoma tissue	
	Number		H‐IME score	*p*	H‐IME score	*p*
e‐AVT	52	AIF RT VS PT	1.01 ± 1.19 versus 2.01 ± 0.87	< 0.0001****	0.45 ± 1.07 versus 1.35 ± 1.25	0.0001***
	25	Non‐AIF RT VS PT	1.24 ± 0.95 versus 1.43 ± 0.91	0.3515	0.81 ± 1.05 versus 1.06 ± 1.08	0.3326
non‐AVT	5	AIF RT VS PT	1.66 ± 0.92 versus 1.48 ± 0.81	0.4047	1.16 ± 1.61 versus 1.28 ± 1.26	0.8565
	24	Non‐AIF RT VS PT	2.47 ± 0.95 versus 1.59 ± 1.04	0.0048**	1.33 ± 1.20 versus 1.23 ± 1.23	0.7951

**p < 0.01; ***p < 0.001; ****p < 0.0001.

In summary, the findings indicate that the H‐IME score based on immune‐related markers, such as immune cells and immune checkpoints, can predict the prognosis of HCC patients. The IME in RT is more immunosuppressive compared to PT, but the immunosuppressive state can be mitigated by e‐AVT and AIF. These findings provide insights into the immunological characteristics of HCC and the potential of AVT and AIF in modulating the immune microenvironment.

## 4 Discussion

In this study, we investigated the number of GrB+ cells, Tregs, and the expression of six immune checkpoints (CTLA‐4, PD‐1, LAG3, TIGIT, TIM3, and VISTA) in tissue samples from HCC patients. We established a H‐IME score to evaluate the IME based on the levels of GrB+ cells, Foxp3+ cells, CTLA‐4, PD‐1, LAG3, and TIGIT. The reliability of the scoring system was validated in an independent set of samples. GrB+ cells represent activated cytotoxic T cells and NK cells, which are the main effector immune cells in the IME responsible for cytotoxic and anti‐tumor activities [31]. Foxp3+ cells represent Tregs, which play an immunosuppressive role in the IME and inhibit the activation and function of effector cells [32]. HBV has the potential to recruit and activate Treg. Compared to viral concerned liver diseases, autoimmune liver diseases have lower level of infiltrating Treg and lower risk of HCC, emphasizing the significance of Treg in inhibitory effect in TIME [33]. Previous studies have shown that the combination of high GrB+ cells and low Foxp3+ cells is a promising independent predictor for OS and RFS in HCC, indicating better prognosis for patients with high GrB+ cell and low Foxp3+ cell infiltration [34]. Additionally, we analyzed the expression of CTLA‐4, PD‐1, LAG3, and TIGIT, which are inhibitory immune checkpoint molecules expressed in immune cells and exert inhibitory effects on the anti‐tumor functions of effector cells by binding to their respective ligands [35]. High expression levels of these checkpoint molecules have been associated with worse prognosis [36, 37, 38]. By integrating these six indicators, namely GrB, Foxp3, CTLA‐4, PD‐1, LAG3, and TIGIT, we developed a comprehensive evaluation system for the IME that takes the number and functional status of infiltrating activated effector cells and inhibitory cells into account. The H‐IME scoring system provides a quantitative assessment of the immunosuppressive state, with higher score indicating a stronger immunosuppressive state.

Our findings revealed that in the non‐AVT group, the IME in l‐RT was more immunosuppressive compared to PT, while no significant change was observed in the IME of e‐RT [39]. In HCC, e‐RT refers to intrahepatic metastasis of the primary tumor, while l‐RT represents recurrence independent of the primary tumor [40, 44]. These two types of recurrence exhibit distinct IME characteristics. Intrahepatic metastases are characterized by an increase in the proportion of CD8+ T cells, but with a memory phenotype and low cytotoxicity; In contrast, true recurrence (recurrence independent of the primary tumor) nodules are infiltrated with cytotoxic and exhausted immune cells [41]. We also observed that the IME of l‐RT tumors were distinct from that of paired PT, showing higher H‐IME scores and more immunosuppressive activities.

Furthermore, we investigated the influence of AVT on the IME. Firstly, we subdivided the AVT group into two categories: e‐AVT and ne‐AVT based on the level of HBV DNA. We found that immunosuppressive activities in the carcinoma and para‐carcinoma tissues of RT were attenuated in the e‐AVT group. It has been reported that AVT could reduce risk of HCC by 70% among patients with chronic hepatitis B [22], and our previous research has demonstrated that AVT improves the prognosis of HCC patients [24]. T cells from patients receiving AVT have been reported to exhibit lower expression of exhaustion‐related genes and higher expression of effector marker genes [42]. Combination therapy with AVT and immune checkpoint inhibitors (ICIs) has shown enhanced anti‐tumor effects, with the upregulation of interferon and Th1 immunostimulatory‐related genes, compared to ICIs alone [43].

Secondly, we compared the IME in the AIF group and the non‐AIF group, stratified by changes in the level of inflammation and fibrosis with help of GS score. Our results showed that the IME in both carcinoma and para‐carcinoma tissues of RT in the AIF group exhibited enhanced anti‐tumor activities. Conversely, the IME in carcinoma tissue of RT cases in the non‐AIF group was characterized by more immunosuppressive activities. HCC is a typical inflammatory‐related tumor that progresses from hepatitis to liver fibrosis, cirrhosis, and eventually HCC [44]. In the chronic phase, inflammation becomes immunosuppressive, characterized by exhausted effector cells and increased infiltration of inhibitory cells such as Tregs； Moreover, macrophages contribute to the activation of hepatic stellate cells and the development of liver fibrosis [45]. Inhibiting fibrosis development can hinder HCC progression [46, 47].

We further observed a significant correlation among e‐AVT, AIF, and IME. Firstly, the proportion of patients with attenuated inflammation/fibrosis levels was higher in the e‐AVT group compared to the non‐AVT group. Secondly, in both the e‐AVT group and the AIF group, the immunosuppressive activities in RT were attenuated compared to PT. Thirdly, H‐IME scores of RT were inferior to those of PT among cases in the AIF group who received e‐AVT, but such change did not happen in cases s in the non‐AIF group who did not receive AVT. These results suggest that e‐AVT may reshape the IME and enhance anti‐tumor activities by relieving inflammation and fibrosis levels. Importantly, the effects of e‐AVT on inhibiting immunosuppressive activities take time to fully manifest [24]. Therefore, we propose a new trilogy corresponding to the traditional trilogy of “hepatitis‐cirrhosis‐HCC” consists of “e‐AVT‐AIF‐ameliorative IME”, corresponding to the traditional trilogy of “hepatitis‐cirrhosis‐HCC”. By inhibiting HBV replication, e‐AVT can ameliorate liver inflammation, stabilize or even reverse fibrosis development, and reshape the IME to enhance local anti‐tumor responses. Renal injury is the most reported side‐effect of AVT, while there were no significant differences between AVT and non‐AVT group in this study. It was reported that tenofovir may lead to more cases of renal injury than entecavir [30], while entecavir was more common in the cohort. Prompt initiation of AVT is crucial for HCC patients with HBV infection, especially with the merge of prospect of cure of HBV [48].

This study has some limitations. Firstly, is the nature of retrospective single‐center study may introduce biases. Secondly, the sample size in the C1 cohort was limited, warranting further investigation of the effects of different AVT regimens on IME, inflammation, and fibrosis. Thirdly, more demographic information and clinicopathological characteristics may contribute to deeply analysis.

In summary, our findings propose a comprehensive scoring system for IME and indicate a close relationship among e‐AVT, AIF, and IME in HCC. e‐AVT can stabilize or even reverse inflammation and fibrosis in HCC, leading to enhanced anti‐tumor activities in the IME. In addition to the traditional trilogy of “hepatitis‐cirrhosis‐HCC,” we propose a new trilogy of “e‐AVT‐AIF‐ameliorative IME”. Early initiation of AVT should be considered for HCC patients with HBV infection to modulate the IME and improve therapeutic outcomes of HCC.

## Author Contributions

**Zhu‐tao Wang:** formal analysis (equal), visualization (equal), writing – original draft (equal), writing – review and editing (equal). **Ruo‐yu Guan:** data curation (equal), formal analysis (equal), investigation (equal), methodology (equal), software (equal). **Wei Gan:** formal analysis (equal), writing – original draft (equal), writing – review and editing (equal). **Zhang‐fu Yang:** investigation (equal), methodology (equal), software (equal). **Bao‐ye Sun:** investigation (equal), methodology (equal), software (equal). **Jing‐fang Wu:** investigation (equal), methodology (equal), visualization (equal). **Dai Zhang:** investigation (equal), methodology (equal), visualization (equal). **Guo‐qiang Sun:** investigation (equal), methodology (equal). **Xu‐kang Gao:** data curation (equal), investigation (equal). **Jin‐long Huang:** writing – original draft (equal). **Gao Liu:** visualization (equal). **Cheng Zhou:** visualization (equal). **Jia Fan:** supervision (equal). **Jian Zhou:** supervision (equal). **Yong Yi:** funding acquisition (equal), supervision (equal), writing – review and editing (equal). **Bo Hu:** conceptualization (equal), funding acquisition (equal), supervision (equal). **Shuang‐Jian Qiu:** conceptualization (equal), funding acquisition (equal), resources (equal), supervision (equal), writing – review and editing (equal).

## Ethics Statement

The study was approved by the Clinical Research Ethics Committee of Zhongshan Hospital. Written informed consent was obtained from all participants.

## Conflicts of Interest

The authors declare no conflicts of interest.

## Supporting information


**Figure S1.** Receiver operating characteristic curves of tumor size, AFP, and H‐IME in cohort C2 (A) and C3 (B).

**Table S1.** Supplementary demographic information in C1 cohort.

**Table S2.** Side‐effect of AVT.

## Data Availability

Data supporting the findings in the study are available upon reasonable request.
