# Supplementary figures and images for: Effective Antiviral Therapy Improves Immunosuppressive Activities in the Immune Microenvironment of Hepatocellular Carcinoma by Alleviating Inflammation and Fibrosis
Source: Cancer Med. 2024 Dec 10;13(23):e70459. doi: 10.1002/cam4.70459 (PMC11632120; doi:10.1002/cam4.70459)

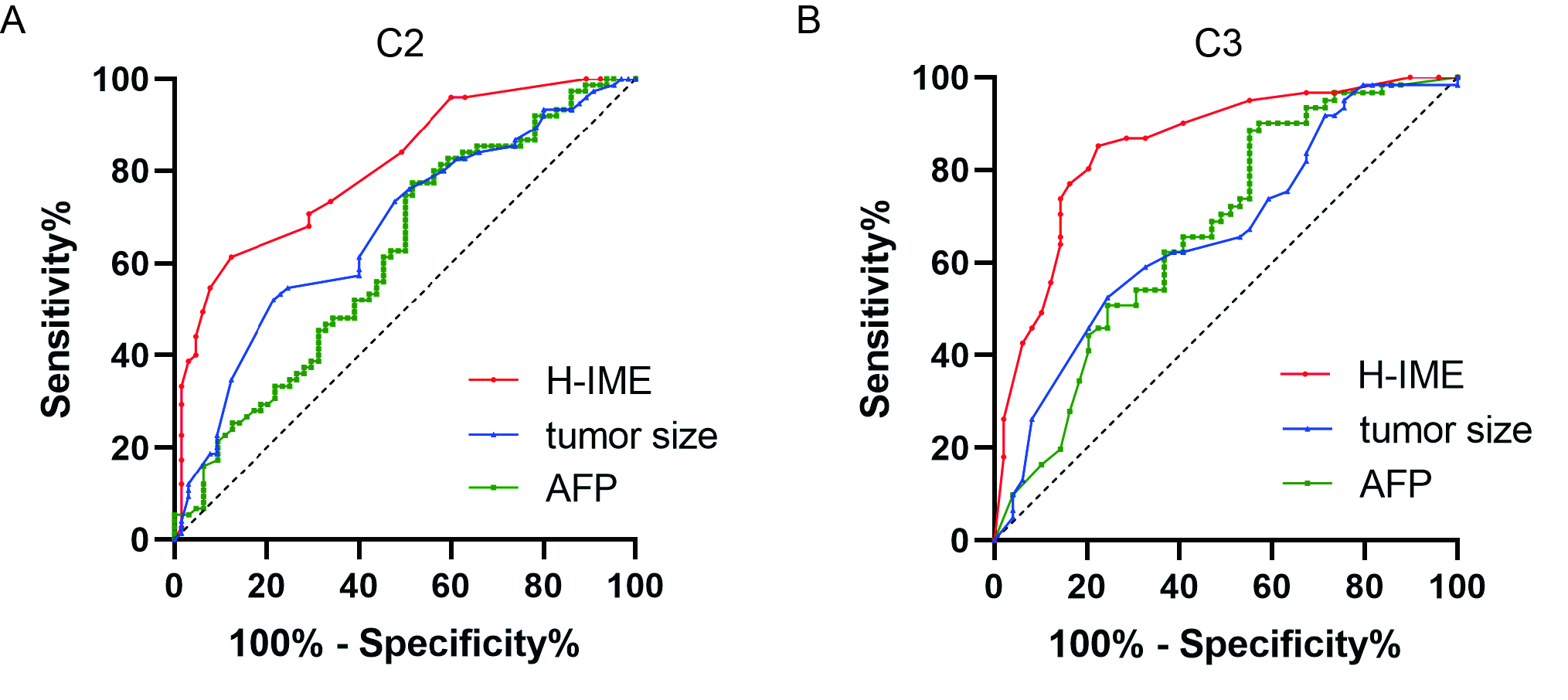

Supplement: Supplementary file 1 — Figure S1. Receiver operating characteristic curves of tumor size, AFP, and H‐IME in cohort C2 (A) and C3 (B). [file CAM4-13-e70459-s003.jpg]
