# Supplementary material for: Effective Antiviral Therapy Improves Immunosuppressive Activities in the Immune Microenvironment of Hepatocellular Carcinoma by Alleviating Inflammation and Fibrosis
Source: Cancer Med. 2024 Dec 10;13(23):e70459. doi: 10.1002/cam4.70459 (PMC11632120; doi:10.1002/cam4.70459)
Supplement: Supplementary file 2 — Table S1. Supplementary demographic information in C1 cohort. [file CAM4-13-e70459-s002.docx]

| **Table S1 Supplementary demographic information in C1 cohort** | |
| --- | --- |
|  | **C1cohort(n=114)** |
| **comorbidities(no/yes)** |  |
| hepatic encephalopathy | 0/114 |
| upper gastrointestinal hemorrhage | 0/114 |
| massive ascites | 0/114 |
| **chronic diseases(no/yes)** |  |
| hypertension | 22/92 |
| diabetes mellitus | 6/108 |
| coronary artery disease | 1/113 |
| **unhealthy lifestyle factor(no/yes)** |  |
| smoking | 24/90 |
| alcoholism | 9/105 |
