# Supplementary material for: Effective Antiviral Therapy Improves Immunosuppressive Activities in the Immune Microenvironment of Hepatocellular Carcinoma by Alleviating Inflammation and Fibrosis
Source: Cancer Med. 2024 Dec 10;13(23):e70459. doi: 10.1002/cam4.70459 (PMC11632120; doi:10.1002/cam4.70459)
Supplement: Supplementary file 3 — Table S2. Side‐effect of AVT. [file CAM4-13-e70459-s001.docx]

| **Table S2 Side-effect of AVT** | | | |  |
| --- | --- | --- | --- | --- |
|  | **AVT** | **non-AVT** | ***P* value** |  |
| **Scr(normal/abnormal)** | 82/3 | 27/2 | 0.4445 |  |
| **BuN(normal/abnormal)** | 81/4 | 28/1 | 0.7752 |  |
| **TBIL(normal/abnormal)** | 79/6 | 28/1 | 0.4843 |  |
| **ALT(normal/abnormal)** | 70/15 | 24/5 | 0.9604 |  |
| Abbreviations: Scr, serum creatinine; BuN, blood urea nitrogen; TBIL, total bilirubin; ALB, albumin; ALT, alanine aminotransferase | | | |  |
|  |  |  |  |  |
